# Epidemiology of Occupational Accidents in Iran Based on Social Security Organization Database

**DOI:** 10.5812/ircmj.10359

**Published:** 2014-01-05

**Authors:** Ramin Mehrdad, Shahdokht Seifmanesh, Farzaneh Chavoshi, Omid Aminian, Nazanin Izadi

**Affiliations:** 1 Center for Research on Occupational Diseases, Tehran University of Medical Sciences, Tehran, IR Iran

**Keywords:** Occupational Accidents, Epidemiology, Iran

## Abstract

**Background:**

Background: Today, occupational accidents are one of the most important problems in industrial world. Due to lack of appropriate system for registration and reporting, there is no accurate statistics of occupational accidents all over the world especially in developing countries.

**Objectives:**

The aim of this study is epidemiological assessment of occupational accidents in Iran.

**Materials and Methods:**

Information of available occupational accidents in Social Security Organization was extracted from accident reporting and registration forms. In this cross-sectional study, gender, age, economic activity, type of accident and injured body part in 22158 registered accidents during 2008 were described.

**Results:**

The occupational accidents rate was 253 in 100,000 workers in 2008. 98.2% of injured workers were men. The mean age of injured workers was 32.07 ± 9.12 years. The highest percentage belonged to age group of 25-34 years old. In our study, most of the accidents occurred in basic metals industry, electrical and non-electrical machines and construction industry. Falling down from height and crush injury were the most prevalent accidents. Upper and lower extremities were the most common injured body parts.

**Conclusion:**

Due to the high rate of accidents in metal and construction industries, engineering controls, the use of appropriate protective equipment and safety worker training seems necessary.

## 1. Background

Occupational accidents are one of the most important consequences of globalization especially in the developing countries (1). The number of fatal occupational accidents in the world has been estimated to be 335000 in 1994 and 350000 in 2001 (1, 2)The number of occupational accidents resulted at least 3 days absence from work has been more than 263,000,000 and 268,000,000 cases in 1998 and 2001 respectively (1). The occupational accidents statistics are published in many countries annually, however because of under-reporting, these data are not so reliable (2). For example in 1998, only 3.9% of worldwide occupational accidents have been reported to International Labor Organization (ILO) (3). In Middle Eastern countries this rate has been estimated to be less than 1% (3). In addition, lack of integrated registration system causes difficulty in comparing these values (2).

Regarding the differences in social status, religion, gender, age distribution of working population and industry sectors among countries, the rates of occupational accidents are very different in the world. It is difficult to generalize statistics related to accidents to other places (4). Despite downward trend of occupational accidents in developed countries, globalization causes increase in occupational accidents in developing countries (1). Accurate occupational accidents registration based on ILO recommendation and analysis of statistics are among the most important tools for implementing preventive strategies (1), (5-7).

Hämäläinen et al. estimated the ratio of occupational accidents in Middle East Countries (MEC) including Iran based upon analysis of data in Turkey, Egypt, Morocco, Tunisia, and Bahrain. No any data directly from Iran was analyzed by Hämäläinen et al. (1, 3, 4). In their study occupational accidents rate has been estimated to be 12845 in 100000 workers in Iran (3). There is no previous national study to describe occupational accidents in Iran. Therefore, despite of reduced access to data, in first step we decided to investigate the characteristics of occupational accidents in Iran according to age, gender, industry, accident type and injured body part.

## 2. Objectives

The aim of this study is epidemiological assessment of occupational accidents in Iran.

## 3. Material and Methods

We analyzed data of occupational accidents recorded by Iranian Social Security Organization (ISSO). The ISSO records occupational accidents’ information through routine documents and computerized system. According to ISSO law, an occupational accident is defined as a situation, occurred in the work time, which leads to physical or mental harm (8). The work time involves the whole time that insured worker performs his or her task in the workplace or sent out of workplace for a mission by employer, during the transportation to and from the workplace and also when worker spend his or her time for a therapeutic purpose. Employers must report the occupational accidents within three working days after it happens to the proper branch of ISSO in special forms and check lists, according to law (8).

Only events that occurred in 2008 were included. In this cross-sectional study, 22158 cases of occupational accident were recorded among 8767638 registered workers of ISSO in 2008. The variables included age, gender, type of industry, type of accident and the injured body part that were collected from documents. SPSS software version 16 was used for analyzing the data.

## 4. Results

**Figure 1.**
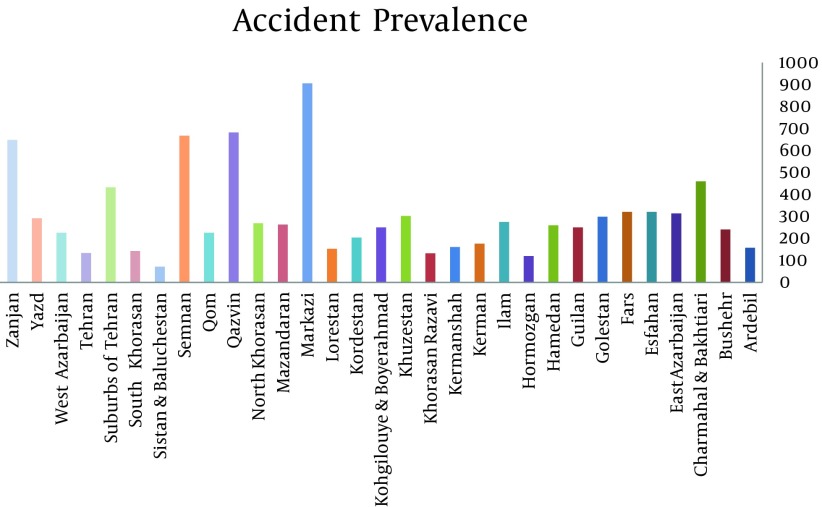
Accident Prevalence in 100,000

Total numbers of 22158 occupational accidents were analyzed among which 20996 cases caused absence from work for 3 days or more. Totally 83 cases were fatal which all occurred among male workers. The fatal and non-fatal accidents ratios are 0.95 in 100,000 and 253 in 100,000 respectively. Accidents ratios among male (21763/7586096) and female (395/1181542) workers were 290 in 100,000 and 30 in 100,000 respectively. Over two-thirds (67.4%) of all accidents occurred in workers aged 34 years or less while the highest percentage of fatal occupational accidents was in the workers older than 55 years. The mean age of injured workers was 32.07 ± 9.12 years which was higher for men (32.12) compared to women (29.25). Table 1 shows the distribution of occupational accidents by age groups. About 24.7% of injured workers were single and 75.3% were married. Provinces had the highest number of incidents were shown in Table 2. In case of accident type, fall from height, crush injuries and fractures had more frequency than other accident types (Tables 3 and 4). Considering injured body parts, upper and lower extremities were more injured. Table 5 presents distribution of occupational accidents by injured body part. Figure 1 shows prevalence accidents in different provinces of Iran

**Table 1.** Occupational Accidents by Age Groups

Age Group	Occupational Accidents, No. (%)	Fatal Occupational Accidents, No. (%)
**15-24**	4399 (19.9)	10 (0.23)
**25-34**	10533 (47.5)	28 (0.26)
**35-44**	4788 (21.6)	24 (0.50)
**45-54**	1910 (8.7)	15 (0.78)
**> 55**	528 (2.3)	6 (1.13)
**All**	22158 (100)	83 (0.95)

**Table 2.** The Highest Prevalence of Fatal and Non-Fatal Occupational Accidents in 100,000 workers in Iran Provinces

Provinces	Occupational Accidents (Prevalence in 100,000)	Provinces	Fatal Occupational Accidents (Ratio)
**Markazi**	905	Semnan	6.20
**Qazvin**	682	Hamedan	5.44
**Semnan**	667	Kordestan	5.26
**Zanjan**	648	Kermanshah	2.60
**Charmahal&Bakhtiari**	460	Markazi	2.10
**Tehran suburb **	432	Khuzestan	1.89
**Esfahan**	321	Kohgilouye and Boyerahmad	1.55
**Fars**	321	Lorestan	1.46

**Table 3.** Shows Distribution of Occupational Accidents by Industrial Sectors

Industrial sector	Occupational Accidents Numbers, No. (%)	Fatal Occupational Accidents, NO. (%)^a^
**Metal industry, electrical and non-electrical machinery**	5398 (24.4)	6 (0.11)
**Construction**	4303 (19.4)	27 (0.63)
**Contractor**	3661 (16.6)	11 (0.30)
**Manufacture of chemicals and chemical products**	2171 (9.8)	3 (0.14)
**Services**	1101 (5)	8 (0.73)
**Manufacture of food and tobacco products**	1063 (4.8)	2 (0.19)
**Mining**	907 (4.1)	6 (0.66)
**Manufacture of wood , paper, tanning, publishing, printing**	865 (3.9)	4 (0.46)
**Manufacture of textiles, wearing apparel and footwear**	744 (3.4)	4 (0.54)
**Transport, storage and communications**	560 (2.5)	7 (1.25)
**Agriculture, hunting, forestry and fishing**	525 (2.4)	0 (0)
**Other sectors**	347 (1.6)	4 (1.07)
**Commercial, bank, insurance**	309 (1.4)	0 (0)

^a^Percent of fatal accidents of each industry sectors

**Table 4.** Occupational Accidents by Type

Accident Type	Frequency, No. (%) CI
**Falling of person**s	4367 (18.4)
**Crush injury**	3169 (13.3)
**Fractures**	2903 (12.2)
**Amputations**	2799 (11.8)
**Caught in or between objects**	2398 (10.1)
**Falling of objects**	2111 (8.9)
**Others**	6009 (25.3)

**Table 5.** Occupational Accidents by Injured Part of the Body

Body Part	Frequency No. (%)
**Head and neck**	2231 (9.4)
**Upper limbs (except fingers)**	5390 (22.8)
**Fingers**	6108 (25.8)
**Trunk**	610 (2.6)
**Lower limbs (except toes)**	6053 (25.6)
**Toes**	653 (2.8)
**Others**	21612 (11)

## 5. Discussion

The present study shows that the rate of occupational accidents in Iran is largely different from results of previous studies (3, 4). Such a difference has been also shown (75663 vs. 3145632) in another study that was conducted in Turkey (3, 9). This difference can be due to using different methods, workforce population and statistical reference population. We considered all recorded accident reports of about 8800000 insured workers of ISSO in 2008 and found 22158 cases while Hämäläinen et al. estimated about 2500000 (2006) and 2800000(2009) occupational accidents in Iran in 1998 and 2001 respectively. However, this difference may be due to possible under-reporting in ISSO. We did not have access to all happened accidents, especially those without significant effects on health and occupational situations. Furthermore, occupational accidents of other insurance companies and self-employed workers don’t record regularly; however the ISSO has insured the maximum number of workers in the industries.

More than two-third of injured workers had age of 34 years or less. This finding is close to other studies (9-11). This fact may be due to less experience and doing more dangerous jobs by young workers (Salminen 2004). In addition, highest percentage of fatal accidents occurred in the workers older than 55 years. This finding is compatible with the previous studies (12, 13). Women constituted less than 2% of injured workers and also their accidents rate was lower than men. This finding has been observed in previous studies (14-16). We should consider that the women employment rates are various in different countries due to economical, religious, industrial and cultural status. Women constituted 13.5% of total employees in the ISSO registry. Also women in Iran employ mostly in less harmful occupations in comparison with men. Thus, this result is expectable and also, is in agreement with the results of Middle East studies (10, 17). Among the occupational accidents, 24.4% belong to the basic metals, 19.4% to the construction and 16.6% to the contractor. This result is not compatible with other studies (Chau, Mur et al. 2004; Liao and Perng 2008; Miguel A. Camino López 2008). Although it is in agreement with Sinana Unsar et al. study in which basic metal and construction sectors had more occupational accidents than other industries (9). Because ISSO didn’t record regularly the number of the insured workers by industrial categories, we couldn’t assess the ratio of occupational accident based on industrial sectors.

Most of the accidents have occurred in central provinces of Iran may be due to concentration of heavy industries in these provinces. Most of fatal occupational accidents were occurred in the west of Iran. This may be due to culture of people that live in these areas (Rejection of preventive education provided by experts in the field of occupational accidents), Small number of experts in occupational health and the high density industries in the west of Iran. In our study, upper and lower extremities constituted about third-fourth of all injured body parts. This result is similar to other studies (18-20).

There are various studies about accident types or accident mechanism around the world. The fall was in the top of the accident type (18.4%) in present study that is in agreement with other studies (14, 15, 21, 22). Our study limitations were: our statistics may be under reporting, because, 1: many workplaces are not covered by social security organization, so their events are not registered in this organization. Another limitation was many accidents; especially minor accidents may not be recorded Due to the high rate of accidents in metal and construction industries, engineering controls, the use of appropriate protective equipment and safety worker training seems necessary.

## References

[1] Hämäläinen Päivi (2009). The effect of globalization on occupational accidents.. Safety Sci..

[2] Takala J (1999). Global estimates of fatal occupational accidents.. Epidemiology..

[3] Hämäläinen Päivi, Takala Jukka, Saarela KaijaLeena (2006). Global estimates of occupational accidents.. Safety Sci..

[4] Hamalainen P, Leena Saarela K, Takala J (2009). Global trend according to estimated number of occupational accidents and fatal work-related diseases at region and country level.. J Safety Res..

[5] Jacinto Celeste, Aspinwall Elaine (2004). A survey on occupational accidents’ reporting and registration systems in the European Union.. Safety Sci..

[6] Nishikitani Mariko, Yano Eiji (2008). Differences in the lethality of occupational accidents in OECD countries.. Safety Sci..

[7] (1996). Recording and Notification of Occupational Accidents and Diseases: An ILO Code of Practice..

[8] Iranian Social Security Organization. (2008.). www.tehran.sso.ir.

[9] Unsar Sinan, Sut Necdet (2009). General assessment of the occupational accidents that occurred in Turkey between the years 2000 and 2005.. Safety Sci..

[10] Fayad R, Nuwayhid I, Tamim H, Kassak K, Khogali M (2003). Cost of work-related injuries in insured workplaces in Lebanon.. Bull World Health Organ..

[11] Jackson LL (2001). Non-fatal occupational injuries and illnesses treated in hospital emergency departments in the United States.. Inj Prev..

[12] Laflamme Lucie (1997). Age-related accident risks among assembly workers: A longitudinal study of male workers employed in the Swedish automobile industry.. J Safety Res..

[13] Salminen S (2004). Have young workers more injuries than older ones? An international literature review.. J Safety Res..

[14] Lin YH, Chen CY, Luo JL (2008). Gender and age distribution of occupational fatalities in Taiwan.. Accid Anal Prev..

[15] Zwerling C, Sprince NL, Ryan J, Jones MP (1993). Occupational injuries: comparing the rates of male and female postal workers.. Am J Epidemiol..

[16] Kelsh MA, Sahl JD (1996). Sex differences in work-related injury rates among electric utility workers.. Am J Epidemiol..

[17] Ergor OA, Demiral Y, Piyal YB (2003). A significant outcome of work life: occupational accidents in a developing country, Turkey.. J Occup Health..

[18] Pransky GS, Benjamin KL, Savageau JA, Currivan D, Fletcher K (2005). Outcomes in work-related injuries: a comparison of older and younger workers.. Am J Ind Med..

[19] Chau N, Mur JM, Benamghar L, Siegfried C, Dangelzer JL, Francais M (2004). Relationships between certain individual characteristics and occupational injuries for various jobs in the construction industry: a case-control study.. Am J Ind Med..

[20] Pollack KM, Agnew J, Slade MD, Cantley L, Taiwo O, Vegso S (2007). Use of employer administrative databases to identify systematic causes of injury in aluminum manufacturing.. Am J Ind Med..

[21] Bhattacherjee A, Chau N, Sierra CO, Legras B, Benamghar L, Michaely JP (2003). Relationships of job and some individual characteristics to occupational injuries in employed people: a community-based study.. J Occup Health..

[22] Macedo AngelaC, Silva InêsL (2005). Analysis of occupational accidents in Portugal between 1992 and 2001.. Safety Sci..

